# Case of Intermittent Cranial Neuralgia, Resulting from Decayed Teeth

**Published:** 1855-07

**Authors:** J. Richardson

**Affiliations:** Cincinnati


### CASE OF INTERMITTENT CRANIAL NEURALGIA, RESULTING FROM DECAYED TEETH.

BY J. RICHARDSON, M. D., D. D. S., CINCINNATI.

We took occasion some time since, through the medium of the "*Western Lancet,*" to direct the attention of medical practitioners to the intimate sympathies, existing between certain structures associated with the teeth, and those of other parts of the system, particularly of the head and face. We also endeavored to point out the influence or agency which a *diseased* condition of these organs had, by virtue of such relations, in the production of other morbid conditions variously located in remote as well as contiguous parts; also the importance of a careful and faithful study of the direct, and undoubted *dependence* which many of these affections may have on some exciting or predisposing morbific influence originally located in, and transmitted from, the teeth.

The peculiar opportunities afforded the members of the dental profession, for observation in this respect, impose upon them a binding obligation to improve them conscientiously, and intelligently, in behalf of afflicted humanity. A proper use of these opportunities will tend to enlarge our sphere of usefulness by a natural, and legitimate co-operation with the members of our sister profession, in a mission of mercy and benevolence. It will add increased dignity, character, and importance to the profession, by opening up the way to a more earnest, and general inquiry into a prolific means of medication in certain classes of diseased action, heretofore illy understood, and illy appreciated, and thus by provoking amendment, secure to suffering patients who are entitled to our best skill, the greatest good by substituting in very many instances, a simple, effective, and rational treatment, for one, that too often, for the credit of medical science, proves entirely impotent.

We are convinced that the importance of this feature in the etiology of many troublesome, and afflictive nervous complaints has heretofore been sadly underrated, and we venture tos affirm, that were the unwritten history of a large proportion of these affections, manifesting themselves under one or other of the multiform aspects of facial, or cranial neuralgia, known, it would force the humiliating conviction upon every mind, that much of the ordinary practice, in these cases is essentially empirical, abortive, and often disastrous.

We do not claim that *all* nervous disorders about the head and face originate from the teeth, even when these organs are in a diseased condition, nor do we insist upon their immediate removal. There are many other causes both local and constitutional quite adequate to the production of similar results. But we *do* insist that where a judicious system of treatment, predicated upon other immediate or remote causes, has been faithfully prosecuted for a reasonable length of time without any material mitigation of symptoms, that reference should be had at once to the condition of the teeth and every useless and offending organ promply removed, followed, if neccssary, by such other treatment as may be deemed best for the restoration of unhealthy gums or processes to their normal condition. Although the extraction of such teeth may not always be thought absolutely necessary as *preliminary* or *introductory* to other treatment, yet we believe that, as a general thing, it should be made so. No bad consequences are likely to result from such a course—much good may accrue. All teeth that are beyond redemption, though they may have no immediate connexion with existing disease in other parts, are continually, though perhaps not appreciably, offensive and pernicious provocators of general ill health. We want no better evidence of this, than the teachings of nature, manifested in her continued efforts to rid herself of these mischievous, and unprofitable members. The same general law is observable throughout the animal economy, and the indication is not to be disregarded but furnishes an unequivocal hint for our edification in these cases.

Although we can offer to the profession but a limited experience in the practice of dentistry, we could, if our limits permitted, furnish a number of cases illustrative of some of the introductory remarks of this paper. We content ourselves for the present, with offering the following, which came to our notice recently.

The case in question, was that of a lady patient, of this city, aged about forty years; at that time (April) in her fifth month of pregnancy.* With the exception of periodical attacks of an asthmatic character which generally recur during the summer months, her general health has been good. During her late affliction she was under the medical care of my brother, Dr. B. F. Richardson, to whom I am partly indebted for the following outline history of her case.

* A very general prejudice prevails in both the medical, and dental professions against the extraction of teeth for women who are *enciente.* Certain general precautions are certainly necessary but the perils of the operation in such cases we think have been somewhat exaggerated. The attending physician in this case, stated to me that in the treatment of a large number of cases requiring the extraction of teeth, he has seldom been deterred by this circumstance unless the patient exhibited a very high degree of nervous impressibility or had previously acquired the habit of aborting, and that he has never had any bad consequences follow the practice.

Prior to the time of her seeking medical assistance, she had suffered more or less with pain in the upper and lateral portions of the head, at one time upon the right side and then shifting to the left, but ultimately becoming more and more intensified in the right parietal region. These distressing symptoms gradually augmented in severity up to the time of her physician's first visit, embracing a period of some six or seven days. Trusting for a time to her own resources she had made diligent application of liniments, fomentations with some available domestic remedies but without any abatement of the symptoms. The pains at first were of a remitting character, comparatively mild through the day, but returning with increased violence every night about 11 o'clock, and continuing with undiminished severity for some six hours, when the paroxysm would again subside, to be renewed the following night, at about the same hour.

In diagnosing the case, the condition of the teeth was consulted, and a number of them found extensively decayed. A suspicion of their mischievous agency induced a recommendation for their immediate removal. As she suffered no toothache nor indeed any unpleasant sensation connected with the teeth, the patient's skepticism of the *utility* of the operation, fortified her natural *dread* of it, to such an extent that she was unwilling at that time to submit to what was conceived by her physician to be a prudent preliminary step in the treatment of her case. Such constitutional remedies therefore as the case seemed to require were given in conjunction with topical applications, among which was a mixture of chloroform and arnica. These remedies were followed after a brief interval by decided portions of sulphate of quinine and extract hyoscyamus during the apyrexia. They promptly arrested the paroxysm and the patient passed a quiet and untroubled night. She was directed, however, to continue the medicine for a stated period, when the case was dismissed. The patient, however, confident of a permanent cure, disregarded the directions given, and neglected to take the medicine, the result of which was, that the paroxysm returned on the following night at the usual hour, and her physician again called to the case. He again recommended the removal of the teeth but was met by the somewhat plausible argument that as the anti-periodic treatment formerly adopted had been followed by such marked relief it would certainly be accompanied by similar results again. He a second time yielded, and the same general treatment was again pursued, and persevered in for three consecutive days, but signally failed to arrest or even mitigate the paroxysms; its only effect this time being to render them more decidedly *intermittent*, and at the same time to increase the exacerbations of pain to such a degree, that they became almost intolerable. These resources failing the removal of whatever decayed teeth she had was peremptorily urged, and the patient at last, disciplined by pain into a more compliant frame of mind, yielded her assent to the operation.

About noon, therefore, of the third day after the recurrence of the paroxysms, at the solicitation of my brother, I extracted, at one sitting, all such teeth as were beyond redemption. These comprised, in the upper jaw, the remains of the right posterior molar, the roots of which were all separated by decay, and the fangs of the anterior bicuspid,—on the left, the roots of the cuspid and posterior bicuspid. These and the left anterior bicuspid which was sound, were all that remained of the upper denture. Below, upon the right, I removed the two bicuspids and upon the left the posterior molar and bicuspids. Most of these teeth were decayed to the gum. There was no other evidences of diseased gums, than the inflamed margins usually surrounding the necks of teeth in that condition. The investments of the fangs, (some of which were partially absorbed,) presented the usual appearances of diseased action, but no sacular abscesses were detected at the apex of any. I was informed that though her neuralgic difficulty was not *accompanied* in the slightest degree by pain in the teeth, yet she had suffered somewhat from tooth-ache a short time prior.

Desirous of testing the therapeutic virtues of the *forceps* in this case, no further treatment was instituted.

The patient was again seen on the following morning. There had been no recurrence of the attack the night before, and the patient has experienced complete immunity from pain or any unpleasant symptoms, up to this time. She expresses great satisfaction at the success of an operation that at once relieved her of a number of decayed and offensive teeth and what would otherwise, in all probability, have proven a most intractable malady.

There is one suggestive feature in the history of this case, that may be profitably remembered. There was no manifest or obvious condition of these teeth, certainly no indication resulting from pain, in any one of them, that would ordinarily lead the mind to associate the lesions in these organs with the remote affection that was subsequently demonstrated, to be purely *sympathetic*. It is therefore neither necessary nor prudent to wait for, or rely upon, such indications, but as in this case, proceed without unnecessary delay upon a *presumption* which gives to the patient a *possible* and in many cases, a *certain* chance of prompt relief.

